# Platelet activation parameters and platelet-leucocyte-conjugate formation in glioblastoma multiforme patients

**DOI:** 10.18632/oncotarget.25395

**Published:** 2018-05-25

**Authors:** Sascha Marx, Maximilian Splittstöhser, Frederik Kinnen, Eileen Moritz, Christy Joseph, Sebastian Paul, Heiko Paland, Carolin Seifert, Madlen Marx, Andreas Böhm, Edzard Schwedhelm, Kerstin Holzer, Stephan Singer, Christoph A. Ritter, Sandra Bien-Möller, Henry W.S. Schroeder, Bernhard H. Rauch

**Affiliations:** ^1^ Department of Neurosurgery, University Medicine Greifswald, Greifswald, Germany; ^2^ Department of Pharmacology, Center of Drug Absorption and Transport (C_DAT), University of Medicine Greifswald, Greifswald, Germany; ^3^ Clinical Pharmacy, Institute of Pharmacy, University of Greifswald, Greifswald, Germany; ^4^ Department of Paediatric Oncology and Haematology, University Medicine Greifswald, Greifswald, Germany; ^5^ Department of Ophthalmology, University Medicine Greifswald, Greifswald, Germany; ^6^ Institute of Clinical Pharmacology and Toxicology, University Medical Center, Hamburg, Germany; ^7^ Institute of Pathology, University Medicine Greifswald, Greifswald, Germany

**Keywords:** platelet activation, glioblastoma multiforme, P-selectin, sphingosine-1-phosphat, PSGL-1

## Abstract

Patients with glioblastoma multiforme (GBM) suffer from an increased incidence of vascular thrombotic events. However, key influencing factors of the primary hemostasis have not been characterized in GBM patients to date. Thus, the present study determines the activation level of circulating platelets in GBM patients, *in-vitro* reactivity to agonist-induced platelet stimulation and the formation of circulating platelet-leucocyte conjugates as well as the plasma levels of the proinflammatory lipid mediator sphingosine-1-phosphate (S1P). The endogenous thrombin potential (ETP) was determined as global marker for hemostasis.

The 21 GBM patients and 21 gender and age matched healthy individuals enrolled in this study did not differ in mean total platelet count. Basal surface expression of platelet CD63 determined by flow cytometry was significantly increased in GBM patients compared to controls as was observed for the concentration of soluble P-selectin in the plasma of GBM patients. While the ETP was not affected, the immunomodulatory lipid S1P was significantly decreased in peripheral blood in GBM. Interestingly, monocyte expression of PSGL-1 (CD162) was decreased in GBM patient blood, possibly explaining the rather decreased formation of platelet-monocyte conjugates.

Our study reveals an increased CD63 expression and P-selectin expression/ secretion of circulating platelets in GBM patients. In parallel a down-modulated PSGL-1 expression in circulating monocytes and a trend towards a decreased formation of heterotypic platelet-monocyte conjugates in GBM patients was seen. Whether this and the observed decreased plasma level of the immunomodulatory S1P reflects a systemic anti-inflammatory status needs to be addressed in future studies.

## INTRODUCTION

The glioblastoma multiforme (GBM) is the most common primary brain tumor in adult patients [1]. Besides a dismal overall survival of this devastating disease, patients with GBM show an increased incidence in venous and arterial embolic events, not only in comparison to healthy humans, but also to most patients with malignancies outside the central nervous system, further limiting prognosis and quality of life of these patients [2–4].

A coagulative potential of malignant diseases was already described by Armand Trousseau over 150 years ago [5]. However, the pathophysiological background in GBM was mainly studied with regard to the plasmatic hemostasis [6–9]. In comparison, the role of the primary hemostasis, i.e. the activation status of circulation platelets is less clear. Hyperreactivity of circulating platelets can be characterized by the expression pattern of surface receptors such as P-selectin [10] and also involves the release of auto- or paracrine activation parameters such as sphingosine-1-phosphat (S1P) [10, 11]. S1P is a versatile immunomodulatory lipid mediator which has been implicated in several inflammatory conditions and malignant diseases including GBM [11, 12]. Platelets are not only the main player of the primary haemostasis, but interact with the immune system also. This is not only reflected by the secretion of immunomodulatory mediators, but also involves formation of heterotypic conjugates between platelets and leucocytes. The formation of such circulating platelet-immune cell conjugates is considered as a surrogate of inflammatory responses and is well described in diseases such as sepsis and atherosclerosis [13–16]. After *in vivo* or *ex vivo* platelet activation, the formation of platelet-leucocyte conjugates (PLC) is typically elevated [10, 16].

The role of platelets in the pathophysiological concept of GBM has been addressed by Brockmann *et al.*, as they have shown a negative correlation between thrombocytosis in the lab prior to surgery and the overall survival in these patients [17–19]. The aim of the present study was to characterize the primary hemostasis in GBM patients by assessing the activation status of circulating platelets and their *ex vivo* reactivity to agonist-induced platelet stimulation. Furthermore, the capacity to form platelet-leucocyte conjugates as well as the circulating S1P levels were assessed. In addition, the calibrated thrombin generation potential was determined as global marker for haemostasis.

## RESULTS

### Study patients

21 consecutive patients (8 female, 13 male, mean age 71 years, range from 55 to 86 years) with newly diagnosed GBM were enrolled in the study. Likewise, the study protocol was applied to 21 age- and gender matched healthy controls (CON) (8 female, 13 male, mean age 65 years, range from 47 to 83 years). Medical history, medication and laboratory parameters are summarized in Table 1. Of note, platelet count did not differ in both groups (GBM: 257 Gpt/l, range from 135 to 383; CON: 245 Gpt/ l, range from 169 to 375), but the GBM-group had a higher total leucocyte blood count. Furthermore, GBM patients received more often dexamethason, levitiracetam, thrombosisprophylaxis with low molecular weight heparin and proton-pump-inhibitors.

**Table 1:** Medical history and laboratory investigations in GBM-patients and controls

	GBM-patients (*n* = 21)	controls (*n* = 21)	*p*-value	statistically significant?
**previous medical history**				
arterial hypertension	15	10	0,21	no
diabetes mellitus	3	0	0,23	no
deep vein thrombosis	2	2	1,00	no
smoking	4	2	0,66	no
**medication**				
antiplatelet drugs	0	0	1,00	no
coumarin-therapy	0	0	1,00	no
low molecular weight heparin	10	2	0,01	yes
paracetamol	6	5	1,00	no
dexamethason for tumor edema	13	0	<0,01	yes
levitiracetam for symptomatic epilepsy	8	0	<0,01	yes
statins	7	3	0,28	no
proton pump inhibitor	18	6	<0,01	yes
**laboratory investigation (mean (range))**				
platelet count [Gpt/l]	257 (135–383)	240 (169–365)	0,50	no
platelet volume [fl]	11 (9,5–12,3)	10,5 (9,6–12,9)	0,10	no
blood leucoyte count [Gpt/l]	12,7 (5,4–21,8)	8,2 (4,5–19,2)	0,02	yes
hemoglobin [mmol/l]	8,4 (7,1–9,4)	8,8 (7,6–10,3)	0,20	no
Quick [%]	94 (21–125)	101 (74–130)	0,26	no
creatinine [μmol/l]	80,6 (66–134)	73,9 (52–114)	0,24	no

Continuous data were analyzed with the Mann–Whitney-*U* Test. Nominal data were analyzed with the Fisher-exact-test. Statistical significant data were assumed with a *p*-value < 0.05.

For determination of the PSGL-1 expression on circulating monocytes, 5 additional consecutive patients (3 female, 2 male, mean age 66 years, range from 48 to 86 years) with newly diagnosed GBM- and 5 age- and gender matched healthy controls (3 female, 2 male, mean age 67 years, range from 54 to 84 years) were enrolled in the study.

### Characterization of the reactivity status of circulating platelets

The evaluation of the platelet activation was performed by quantification of the surface expression of several receptors (CD63, P-selectin, CD40L and fibrinogen binding to the activated GPIIb/ IIIa) immediately after blood sampling by flow cytometry. Since P-selectin is both, expressed on the platelet surface and also released into the peripheral circulation, whole P-selectin was determined by ELISA in plasma as well.

**Figure 1:**
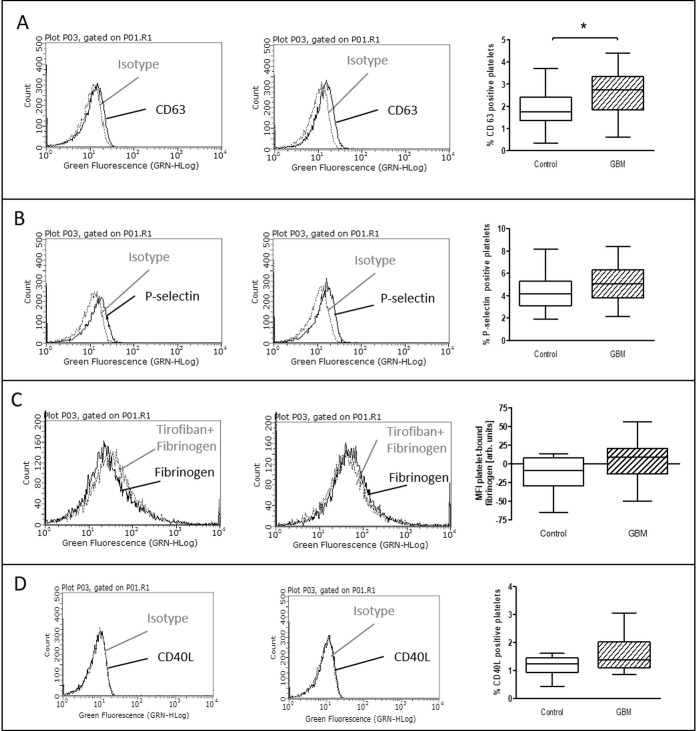
Baseline activation status of platelets in the blood of GBM patients and control individuals The surface expression of CD63 (**A**), P-selectin (**B**) and CD40L (**C**) as well as the specific fibrinogen-binding to the activated GPIIb/IIIa (**D**) was assessed by flow cytometry immediately after blood withdrawn. The results are expressed as the relative numbers of platelets that were positive for CD63, P-selectin or CD40L. The specific fibrinogen-binding is expressed as the median fluorescence intensity (MFI). Histograms containing isotype control (grey line) and specific staining (black line) for control individuals (left column) and GBM patients (middle column) are shown as example. Data are shown as box plots representing the median as horizontal bars as well as the whiskers representing the minimum and maximum (right column). GBM and controls have been each *n* = 19 (A), *n* = 21 (B), *n* = 12 (C) and *n* = 11 (D). Statistical analysis was done with the Mann–Whitney *U* test, ^*^p < 0.05.

**Figure 2:**
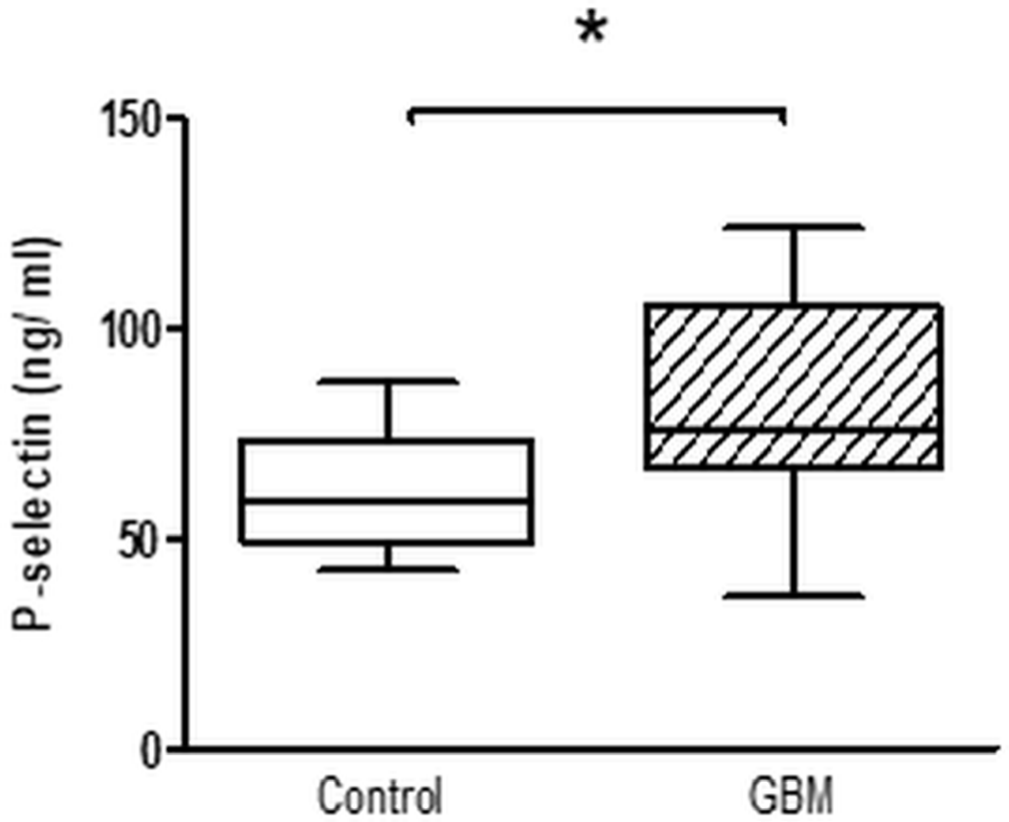
Whole platelet P-Selectin concentration in platelet rich plasma of GBM patients and control individuals After blood sampling, platelet rich plasma was prepared and total P-selectin concentration was assessed by ELISA. Data are shown as box plots with median (horizontal bars) and whiskers (minimum and maximum). *n* = 19 for both control and GBM. Statistical analysis was done with the Mann–Whitney *U* test, ^*^p < 0.05.

The expression of surface activation markers was increased in GBM patients. CD63 expression on platelets in GBM patients was significantly increased by about 50% (GBM: median 2.7%, range from 0.6% to 4.4%/CON: median 1.8%, range from 0.3% to 3.7%/*p* = 0.01) (Figure 1A). Expression of platelet P-selectin was increased by about 25% in GBM patients (GBM: median 5.1%, range from 2.2% to 8.4%/CON: median 4.2%, range from 1.9% to 8.1%/*p* = 0.14), but this was not statistically significant (Figure 1B). However, total plasma P-selectin as determined by ELISA, was significantly increased in GBM patients (GBM: 75.9 ng/ ml, range from 30.6 to 135.6/CON: 58.6 ng/ ml, range from 31.2 to 96.6/*p* = 0.01) (Figure 2). In addition, a trend towards an elevated CD40L expression on the platelet surface was observed in GBM patients (GBM: median 1.4%, range from 0.9% to 3.1%/CON: median 1.2%, range from 0.4% to 1.6%/*p* = 0.10) (Figure 1D). Furthermore, *ex vivo* specific fibrinogen-binding was determined to assess the activation level of GPIIb/IIIa in platelets from GBM patients and controls. Specific fibrinogen-binding, which was determined as difference between fibrinogen binding alone and in the presence of tirofibane as surrogate marker for GPIIB/IIIa activation, was tentatively increased in GBM (GBM: median 8.7 MFI, range from −50.1 MFI to 56.5 MFI/CON: median −8.6 MFI, range from −64.8 MFI to 13.9 MFI/*p* = 0.1), but did not reach statistical significance (Figure 1C).

### Agonist induced platelet activation *in-vitro*

The evaluation of agonist induced platelet reactivity *in-vitro* was performed by using either ADP or TRAP (thrombin-receptor activation peptide), both being potent platelet agonists. Agonists were incubated in whole blood within 45 to 60 min after sampling. Surface expression of several platelet receptors (CD63, P-selectin, CD40L, fibrinogen binding to the activated GPIIb/ IIIa) were quantified by flow cytometry.

**Figure 3:**
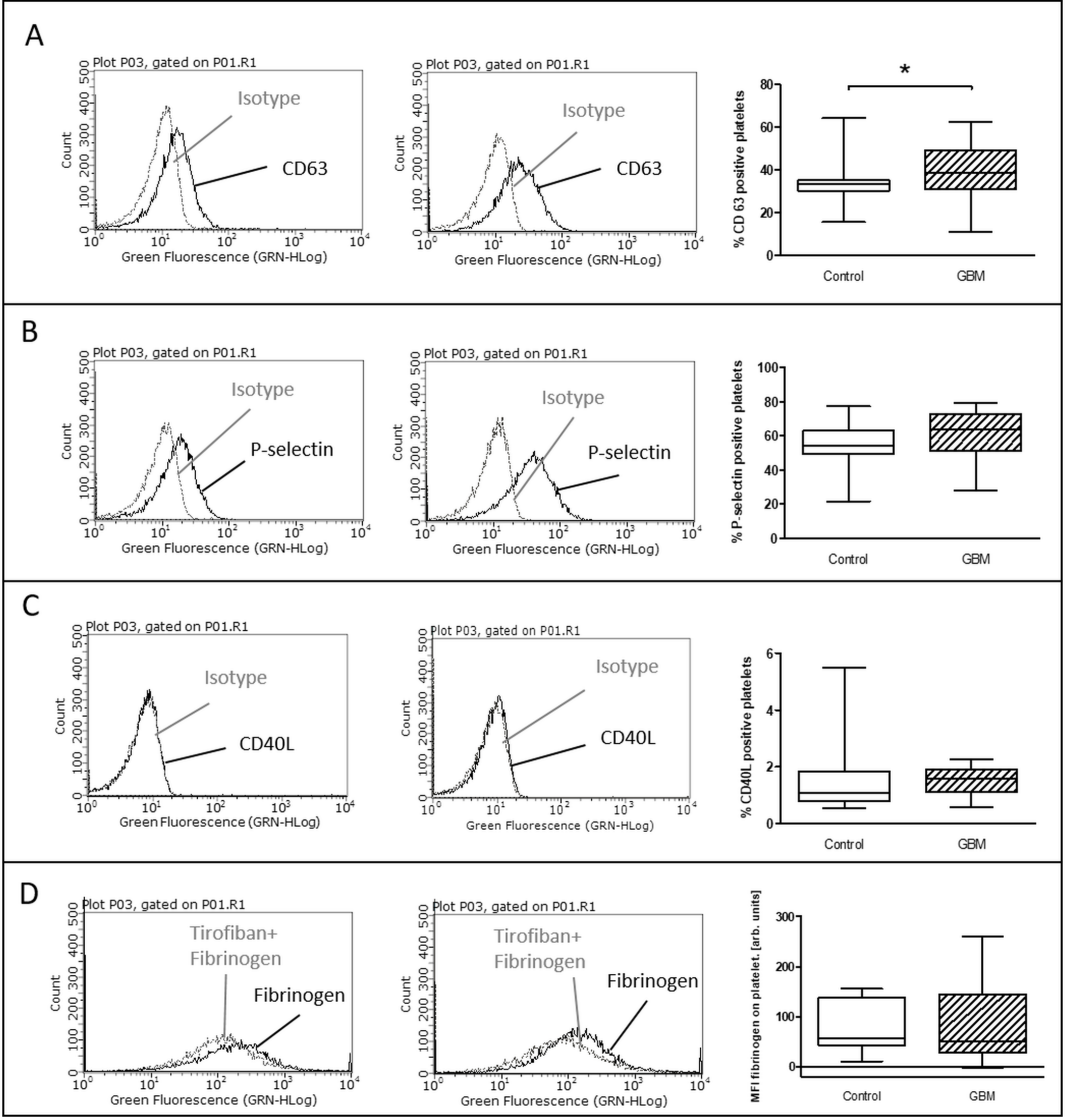
*Ex vivo* stimulation with TRAP in glioblastoma patients and control individuals The surface expression of CD63 (**A**), P-selectin (**B**) and CD40L (**C**) as well as the specific fibrinogen-binding to the activated GPIIb/IIIa (**D**) was assessed by flow cytometry after *ex vivo* agonist platelet stimulation with TRAP. The results are expressed as the relative numbers of platelets that were positive for CD63, P-selectin or CD40L. The specific fibrinogen-binding is expressed as the median fluorescence intensity. Histograms containing isotype control (grey line) and specific staining (black line) for control individuals (left column) and GBM patients (middle column) are shown as example. Data are shown as box plots representing the median as horizontal bars as well as the whiskers representing the minimum and maximum (right column). GBM and controls have been each *n* = 19 (A), *n* = 21 (B), *n* = 12 (C) and *n* = 11 (D). Statistical analysis was done with the Mann–Whitney *U* test, ^*^p < 0.05.

CD63 expression which is classically associated with delta-granule secretion [10] was significantly increased after TRAP stimulation in GBM patients (GBM: median 38.4%, range from 11% to 62.5%/CON: 33.3%, range from 15.5% to 64.4%/*p* = 0.04) (Figure 3A). In comparison, for platelet surface expression of P-selectin only a trend towards an elevation was seen after TRAP stimulation in GBM (GBM: 63.5%, range from 28% to 79.4%/CON: 54%, range from 21.5% to 77.1%/*p* = 0.07) (Figure 3B). Expression of CD40L and the specific binding of fibrinogen to activated GPIIb/IIIa showed no difference between both groups after TRAP-stimulation (Figure 3C, 3D).

**Figure 4:**
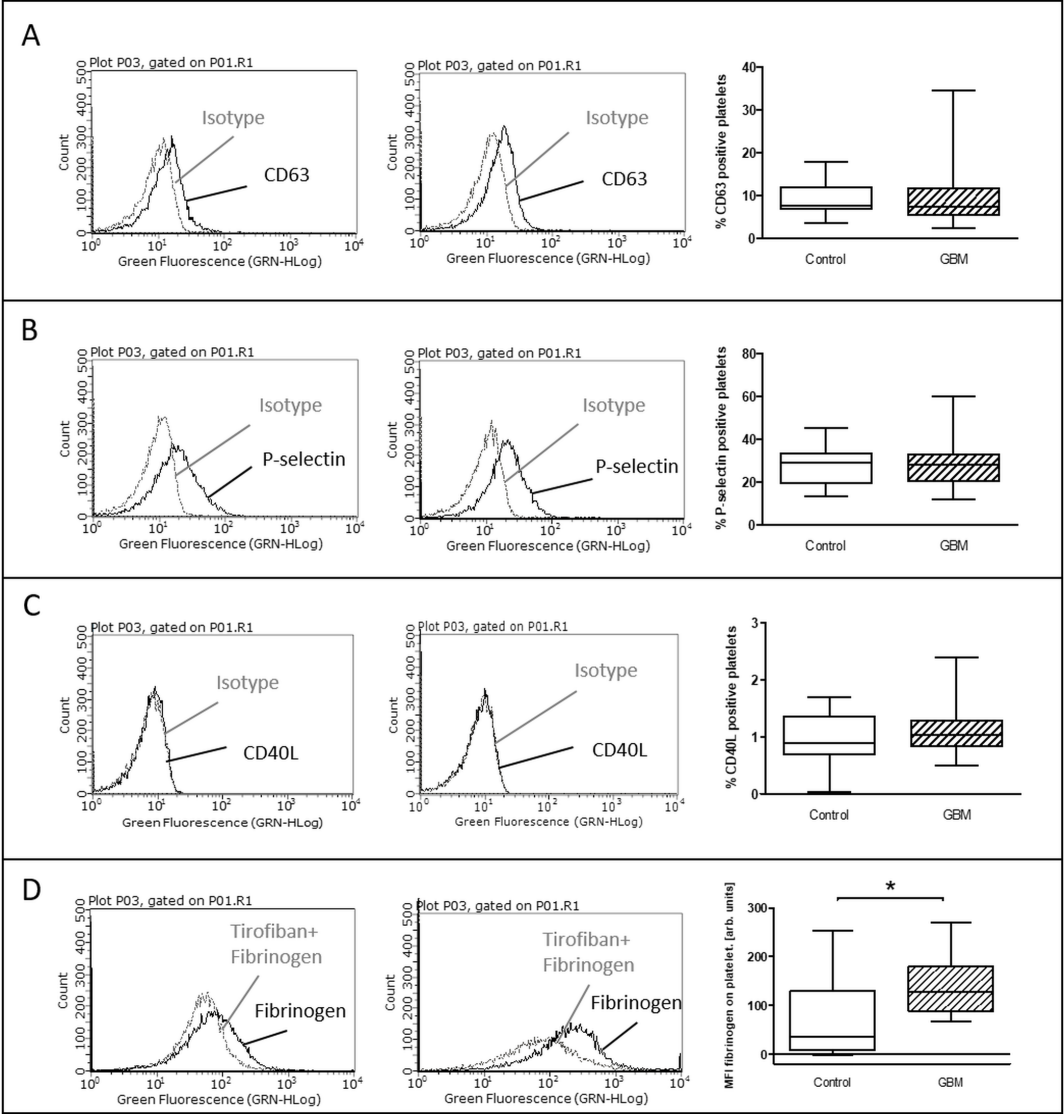
*Ex vivo* stimulation with ADP in glioblastoma patients and control individuals The surface expression of CD63 (**A**), P-selectin (**B**) and CD40L (**C**) as well as the specific fibrinogen-binding to the activated GPIIb/IIIa (**D**) was assessed by flow cytometry after *ex vivo* agonist platelet stimulation with ADP. The results are expressed as the relative numbers of platelets that were positive for CD63, P-selectin or CD40L. The specific fibrinogen-binding is expressed as the median fluorescence intensity. Histograms containing isotype control (grey line) and specific staining (black line) for control individuals (left column) and GBM patients (middle column) are shown as example. Data are shown as box plots representing the median as horizontal bars as well as the whiskers representing the minimum and maximum (right column). GBM and controls have been each *n* = 19 (A), *n* = 21 (B), *n* = 12 (C) and *n* = 11 (D). Statistical analysis was done with the Mann–Whitney *U* test, ^*^p < 0.05.

After *in vitro* incubation with ADP, the stimulated expression levels of CD63, P-selectin and CD40L showed no difference between control and GBM patients (Figure 4A–4C). However, the proportion of specifically bound fibrinogen was significantly increased in GBM patients after *in vitro* stimulation with ADP (GBM: median 128 MFI, range from 67 to 270/CON: median 36, range from −2.6 to 253/*p* = 0.03) (Figure 4D).

### Formation of heterotypic platelet-leucocyte conjugates

**Figure 5:**
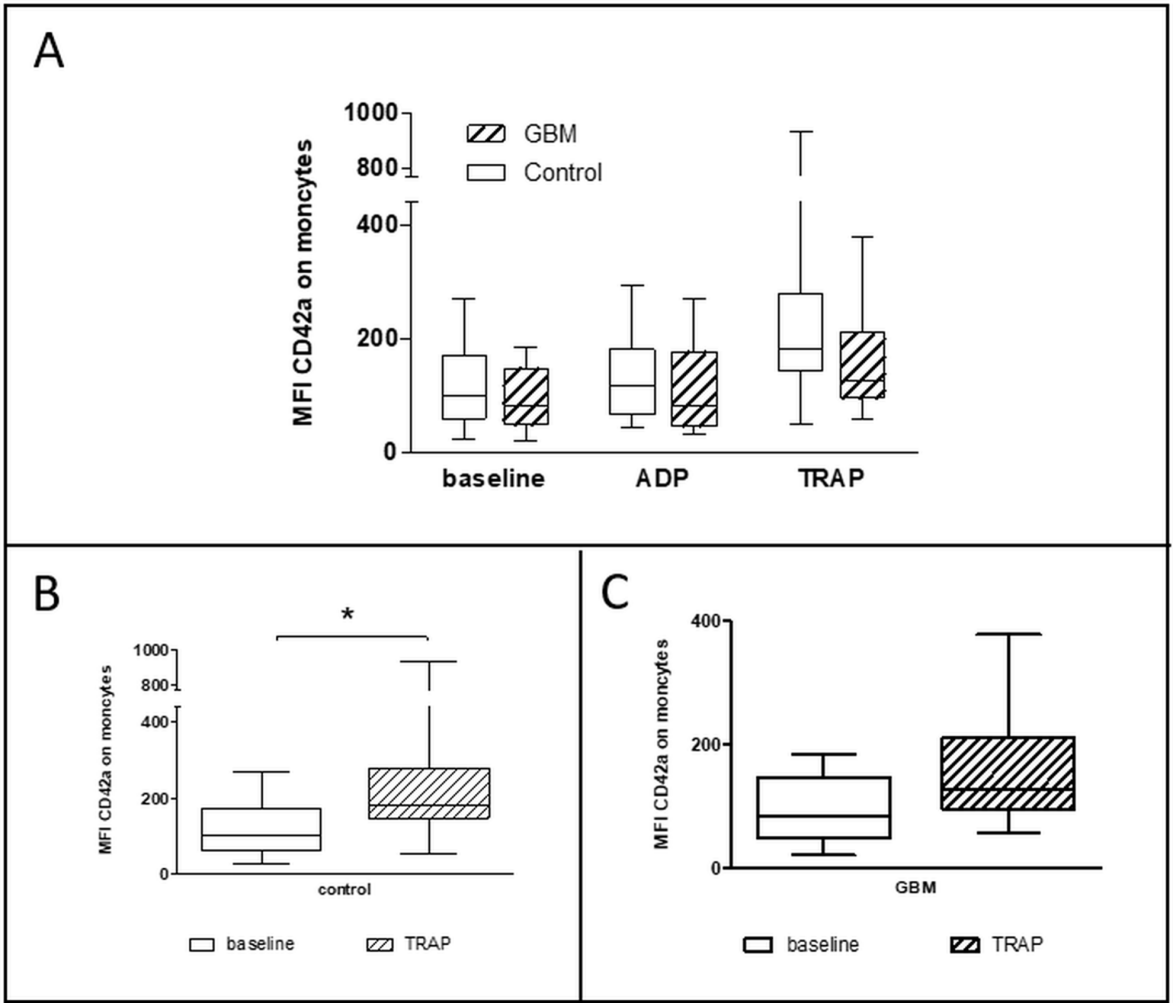
Platelet-monocyte conjugate formation in the circulation of GBM patients and control individuals and after *ex vivo* platelet stimulation with either ADP or TRAP The platelet-monocyte conjugate formation was assessed in glioblastoma patients and controls by flow cytometry immediately after blood withdrawn or after *ex vivo* agonist platelet stimulation with either ADP or TRAP (**A**). The comparison between the basal conjugate formation and the formation after *ex vivo* stimulation with TRAP is shown for controls (**B**) and GBM patients (**C**). Monocytes were defined by the scatter characteristics and CD45 expression. The results are expressed as the median fluorescence intensity of the platelet specific antigen CD42a over the monocyte population. Data are shown as box plots representing the median as horizontal bars as well as the whiskers representing the minimum and maximum. GBM and controls have been each *n* = 14. Statistical analysis was done with the Mann–Whitney *U* test, ^*^p < 0.05.

The formation of heterotypic platelet-leucocyte conjugates (PLC) was analyzed after lysis of red blood cells by flow cytometry. PLC were evaluated immediately after blood sampling and after *in vitro* agonist platelet stimulation with either ADP or TRAP. Platelet-monocyte conjugates (PMC) showed no statistically relevant difference between both groups at baseline (GBM: median 84.1 MFI, range from 21.7 MFI to 185 MFI/CON: median 102.1 MFI, range from 24 MFI to 271 MFI/*p* = 0.39) and after *in vitro* platelet stimulation with ADP (GBM: median 82.3 MFI, range from 32.6 MFI to 271 MFI/CON: median 117.3 MFI, range from 44 MFI to 294 MFI/*p* = 0.28). However, the formation of PMC was clearly and almost statistically significant reduced in GBM patients after *in vitro* platelet stimulation with TRAP (GBM: median 128.5 MFI, range from 59 MFI to 378 MFI/CON: median 182.3MFI, range from 50 MFI to 935 MFI/ *p* = 0.06) (Figure 5A). In comparison, formation of PMC was significantly increased after *in vitro* platelet TRAP stimulation compared to baseline values in controls (*p* = 0.01), but not in GBM patients (*p* = 0.10) (Figure 5B, 5C).

**Figure 6:**
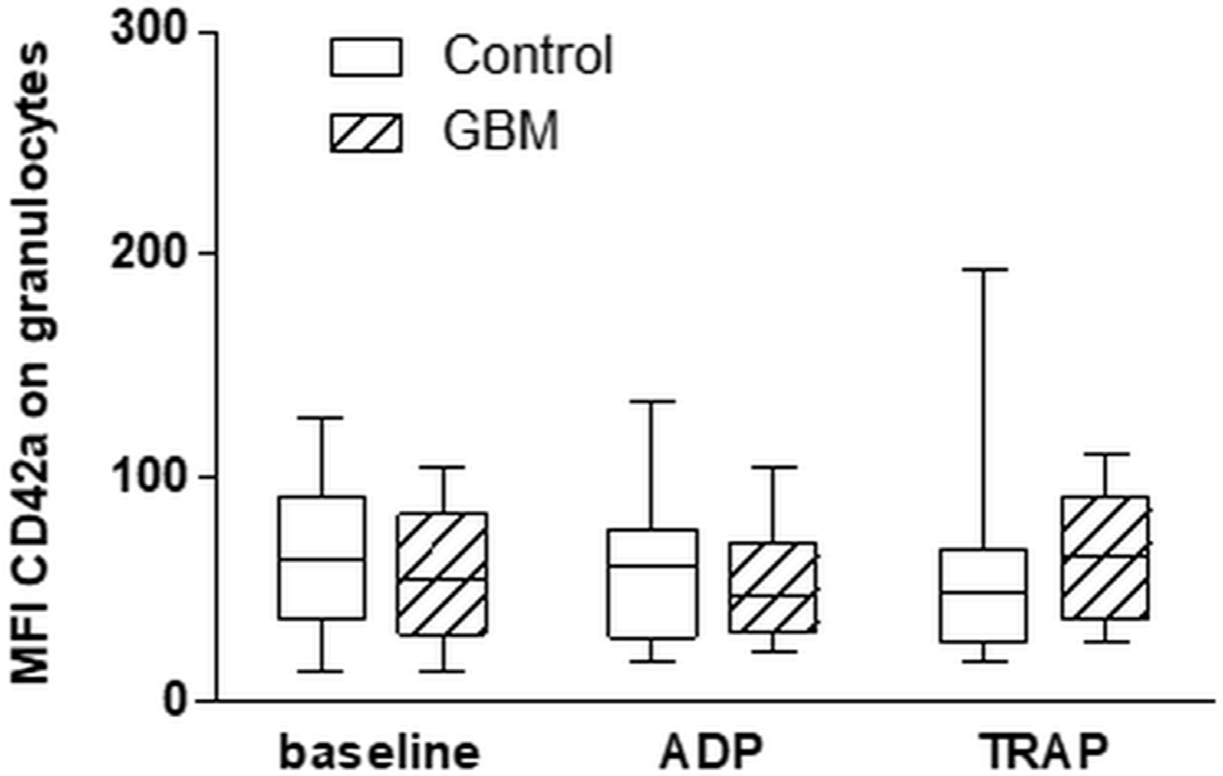
Platelet-granulocyte conjugate formation in the circulation in GBM patients and control individuals and after *ex vivo* platelet stimulation with either ADP or TRAP The platelet-granulocyte conjugate formation was assessed in glioblastoma patients and controls by flow cytometry immediately after blood withdrawn or after *ex vivo* agonist platelet stimulation with either ADP or TRAP. Granulocytes were defined by the scatter characteristics and CD45 expression. The results are expressed as the median fluorescence intensity of the platelet specific antigen CD42a over the granulocyte population. Data are shown as box plots representing the median as horizontal bars as well as the whiskers representing the minimum and maximum. GBM and controls have been each *n* = 14. Statistical analysis was done with the Mann–Whitney *U* test, ^*^p < 0.05.

The formation of platelet-granulocyte conjugates (PGC) showed no statistically relevant differences between GBM patients and controls, neither at baseline nor after platelet stimulation *in vitro* with either ADP or TRAP (Figure 6). The formation of platelet-lymphocyte conjugates was very low and no differences could be detected between both groups (data not shown).

### Automated calibrated thrombin generation

The evaluation of dynamic thrombin generation was performed after thawing of plasma which was frozen immediately after blood sampling. No differences could be detected between both groups with regard to the overall thrombin generation, the peak thrombin generation and the lag time (data not shown).

### Levels of circulating sphingosin-1-phosphat

**Figure 7:**
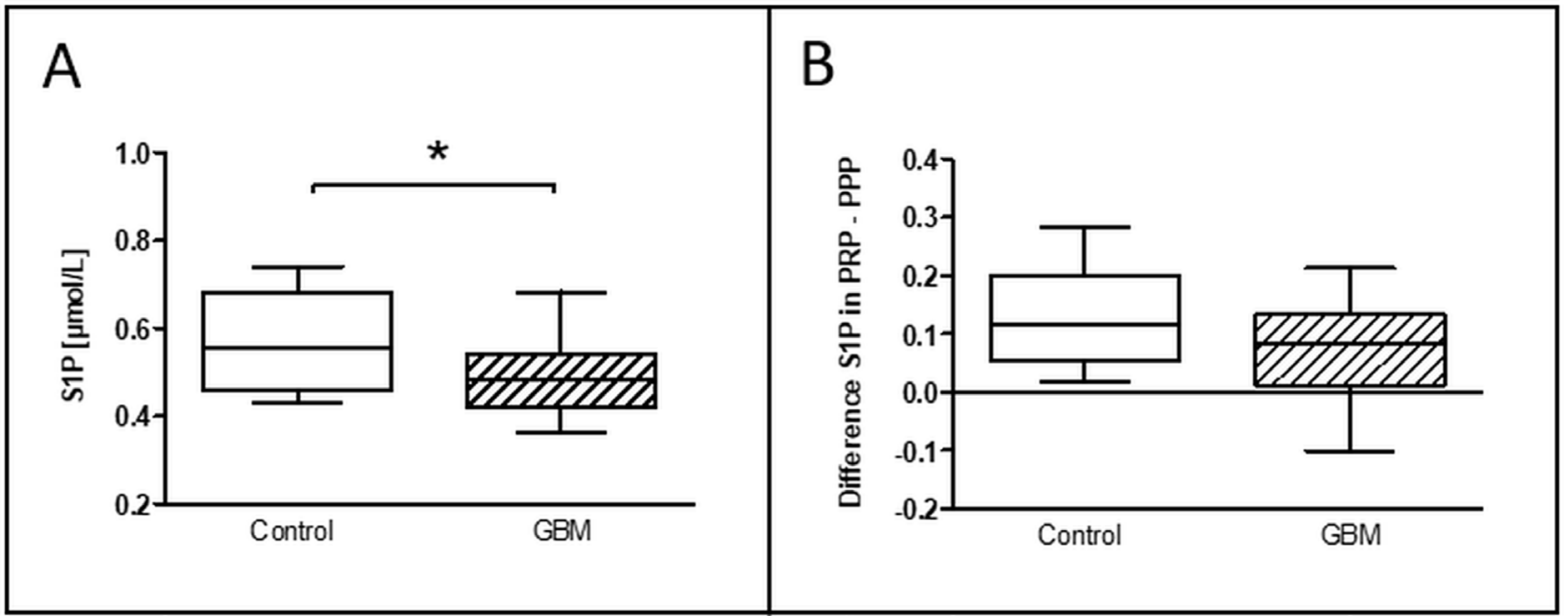
Level of S1P in the circulation of glioblastoma patients and control indivituals The concentration of the circulating level of sphingosine-1-phosphate (S1P) in platelet-rich-plasma was assessed in glioblastoma patients and controls (**A**). The difference between the S1P level in platelet-rich-plasma and platelet-poor plasma is shown as well (**B**). Data are shown as box plots representing the median as horizontal bars as well as the whiskers representing the minimum and maximum. GBM and controls have been each *n* = 19. Statistical analysis was done with the Mann–Whitney *U* test, ^*^p < 0.05.

The evaluation of the circulating level of sphingosine-1-phosphat (S1P) has been measured after thawing of immediately after blood withdrawn frozen platelet rich (PRP) and platelet poor plasma (PPP). Intriguingly, S1P concentration (μmol/ l) in PRP was significantly reduced in GBM patients (GBM: median 0.49, range from 0.36 to 0.69/CON: median 0.56, range from 0.41 to 0.81/*p* = 0.04) (Figure 7A). While no significant differences were detected in PPP between both groups (data not shown), the difference between S1P concentrations in PRP and PPP was almost significantly reduced in GBM patients compared to controls (GBM: 0.09, range from −0.11 to 0.21/CON: 0.12, range from 0.006 to 0.29/*p* = 0.06) (Figure 7B). This may point to diminished platelet S1P release in GBM patients and could also reflect elevated systemic circulating platelet activation.

### PSGL-1 expression on peripheral circulating monocytes

**Figure 8:**
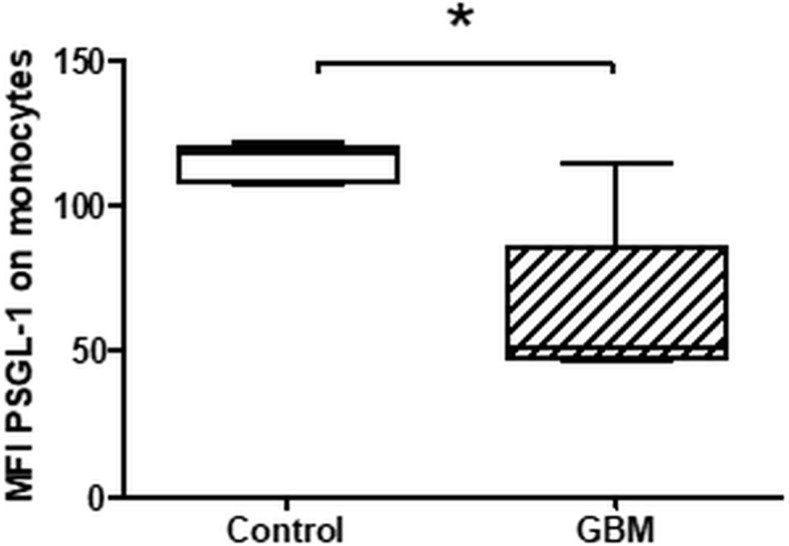
Expression levels of PSGL-1 on circulating monocytes in GBM patients and control individuals The surface expression of PSGL-1 was assessed by flow cytometry. The results are expressed as the median fluorescence intensity (MFI). Data are shown as box plots representing the median as horizontal bars as well as the whiskers representing the minimum and maximum. GBM and controls have been each *n* = 5. Statistical analysis was done with the Mann–Whitney *U* test, ^*^p < 0.05.

According to the paradoxical results of an increased platelet P-selectin secretion and expression and a reduced formation of platelet-monocyte conjugates, we determined the PSGL-1 expression on circulating monocytes in GBM and controls. PSGL-1 expression was significantly reduced in GBM patients (GBM: median MFI 51, range from 46.3 to 114.4; CON: median MFI 117.5, range from 107 to 121.4; *p* = 0.03) (Figure 8). This finding is in agreement with the observed reduced levels of circulating PMC and might reflect an impaired immunological response in GBM patients.

## DISCUSSION

### Summary of the key results

The present study reveals an increased CD63 expression and P-selectin expression/secretion of circulating platelets in GBM patients. Paradoxically, the formation of heterotypic platelet-monocyte conjugates was tentatively decreased in GBM patients after TRAP stimulation. The observed accompanied reduced expression of PSGL-1 on circulating monocytes might provide a possible explanation for this finding. Furthermore, the plasma S1P levels were decreased in GBM patients, while the overall thrombin generation potential as a measure for an elevated capacity to convert prothrombin into active thrombin was not increased.

### Increased activation of circulating platelets in glioblastoma patients

P-selectin is the most studied platelet activation receptor and its elevation on the platelet surface and also its release into the circulation has been shown for several diseases with a thrombotic or inflammatory background such as acute venous thromboembolism, coronary artery disease, stroke, chronic thromboembolic pulmonary hypertension, infectious respiratory diseases and metabolic syndrome [20–25]. Interestingly, P-selectin expression on platelets was reduced in patients with atrial fibrillation compared to healthy controls. However, circulating P-selectin was increased in the plasma of the same patients and the total platelet count was reduced, which may reflect a state of platelet exhaustion and consumption [26]. Increased platelet activation, measured by the P-selectin and GPIIb/IIIa expression has also been shown for patients with myeloproliferative disorders and a mixed population of solid cancers, including malignancies from in- and outside the central nervous system [27, 28].

The observed elevated platelet activity in GBM patients in the present study was most evident by secretion of specific platelet activation markers such as CD63 and P-selectin. The specific fibrinogen-binding to GPIIb/IIIa as surrogate for platelet aggregation was at least not significantly increased in the here investigated GBM patients. This may be due to a high standard deviation in these clinical samples. Similar observations were also reported by others [23]. Expression of CD40L on platelets was not studied so far in malignant diseases and was tentatively increased in the GBM group in our study. The difference between the S1P level in platelet rich and platelet poor plasma was nearly significantly reduced in GBM patients, representing further evidence for elevated platelet activity in GBM patients [11].

### Hyperreactivity of platelets in glioblastoma patients after *in vitro* agonist induced stimulation

In the *in vitro* experiments, platelets were selectively stimulated with either ADP, which is a strong promoter of platelet aggregation, or TRAP, which acts mainly via PAR-1 and is a strong promoter of the secretory function of platelets [29, 30]. Interestingly, *in vitro* stimulation with ADP lead to an increased fibrinogen-binding to the activated GPIIb/IIIa and *in vitro* stimulation with TRAP resulted in a statistically significant increased CD63 and tendentially increased P-selectin expression in GBM patients. In a mixed group of solid cancer patients Riedl *et al.* have shown a diminished GPIIb/IIIa expression after *in vitro* agonist platelet stimulation [27]. However, patients with GBM have not been analyzed separately in this study. In line with our results, Cooke *et al.* have shown an increased reactivity to agonist induced platelet stimulation (measured by P-selectin expression and platelet aggregometry) in patients with metastatic cancer of different origins [31].

There were no differences in the CD40L expression after *in vitro* stimulation between platelets of GBM and healthy controls in our experiments. CD40L expression is known to be less stable and it´s expression decreases as soon as one minute after platelet activation [32]. Although our sample preparation was straight forward, it took about an hour between *ex vivo* agonist induced platelet stimulation and fixation of the samples, which could be an explanation for the missing difference in CD40L expression between both groups in our experiments. Overall, the differences between GBM-patients and healthy control individuals have not been that obvious in the *in vitro* experiments using ADP or TRAP as strong platelet agonists. This finding may point to a latent state of elevated platelet activation in the circulation of GBM patients rather than a predominant effect on platelet activation under maximal agonistic conditions as induced by the incubation with ADP or TRAP *in-vitro*.

### Decreased levels of both circulating S1P and platelet-monocyte conjugates as indications for a systemic anti-inflammatory status?

The formation of conjugates between platelet and leucocytes is well known as surrogate of an inflammatory reaction and described in respective diseases like sepsis and atherosclerosis [16, 33, 34]. The interaction is initiated by P-selectin on platelets and PSGL-1 on leucocytes [10, 15]. Furthermore, the platelet GPIIb/IIIa contributes to the formation process as well [35]. The conjugate formation has been described as most pronounced in the group of monocytes and least in the group of lymphocytes, [36] as also seen in our experiments. Typically, the formation of PLC is increased after platelet activation. This was reflected by a significant increase in the formation of platelet-monocyte conjugates after *in vitro* TRAP stimulation in the control group. However, this was not observed in the GBM group. Although we have seen an increased expression of P-selectin and the fibrinogen-binding to the GPIIb/IIIa on platelets of GBM at baseline and after *in vitro* platelet stimulation, the formation of platelet-monocyte conjugates was rather decreased in GBM patients, which was most pronounced after the strong *in vitro* stimulation of platelets with TRAP. An increased PLC formation has been shown in the circulation of patients with acute venous thromboembolism, metabolic syndrome, coronary artery disease, diabetes mellitus, sepsis and myeloproliferative disorders [14, 20, 21, 28, 37, 38]. An increased P-selectin expression was in agreement with an increased PLC formation in these studies. Riedl *et al* found an increased PMC formation in the blood of a mixed population of cancer patients [27]. However, no separate analysis with regard to GBM was done in this study.

A decreased PLC formation has also been shown in the circulation of atrial fibrillation [26]. Since P-selectin expression on platelets was reduced in these patients, the “failure” to form PLC is most likely platelet-dependent. We have shown an increased P-selectin expression, but not an increased rate of PMC in GBM patients. This appears to point towards a monocyte-dependent cause of reduced PMC formation and is further supported by the finding of a reduced expression level of PSGL-1 on circulation monocytes. Interestingly, the predominant immune cells in the GBM microenvironment are microglia and invading tumor-associated macrophages (TAMs). TAMs exhibit an alternative macrophage phenotype, reflecting a highly immunosuppressive and anti-inflammatory GBM microenvironment [39–41]. The here shown reduced capacity of PMC conjugate formation might represent a circulating surrogate of these intratumoral TAMs, which however can finally not be proven with the data of the present study.

Regulatory T cells, another potential circulating cellular indicator of anti-inflammation, were not found to be elevated in GBM patients [42]. However, T cells are the minority of immune cells in the GBM microenvironment. Another hint for a systemic anti-inflammation in the GBM patients in our study is the reduced level of S1P [11]. In a previous study, we have shown that the S1P receptor- 1 and -2 and S1P metabolizing enzymes are dysregulated in GBM patients and partially correlate with survival time [12]. Since S1P directs immune cell migration via concentration gradient-dependent mechanisms [43], a reduced circulating and in turn elevated S1P level in GBM tissue [44] might foster monocyte migration from the peripheral blood into the brain. In addition, S1P has been reported as a key player in the formation of TAMs via S1P receptor-1 [45]. Thus, reduced peripheral S1P levels could drive monocyte invasion and elevated S1P within the GBM consecutively supports TAM formation.

### Translation relevance of an increased platelet activation in GBM patients

The present study for the first time describes an increased expression of CD63 and increased expression/ secretion of P-selectin in a homogeneous cohort of first diagnosed glioblastoma patients. This observation provides further evidence for the tremendous coagulative potential of malignant tumors and particularly malignant glioma which has been observed in previous clinical studies [4, 5, 27, 46]. Enhanced platelet reactivity to *in vitro* platelet activation is associated with an increased risk of venous and arterial thrombosis [10, 47, 48]. An elevated incidence of DVT in cancer patients, known as Trousseaus phenomenon, was described in the 19th century and is associated with a poor prognosis in cancer patients [5, 49–52]. Amongst different cancer types, patients with malignant gliomas have the highest risk for DVT and reports from the literature are given between 15% and up to 30% of patients [3–5, 46, 53–55]. Furthermore, patients with GBM suffer from an increased incidence of arterial embolic events [2]. Platelets not only contribute to arterial embolic events, but also participate in the pathogenesis of DVT [21, 56, 57].

In contrast to the suspected connection between increased platelet activation and an increase of vascular events, Riedl *et al.* have shown that a diminished P-selectin and GPIIb/IIIa expression on platelets was associated with a higher risk of DVT in a mixed cohort of cancer patients from different sides [27]. A proper explanation would be an exhausted stage of platelets in a highly pro-thrombotic environment. Exhausted platelets have previously been shown in a mixed group of cancer patients [58] Although circulating tumor cells are detectable at a low level in GBM patients as well, GBM virtually never perform metastasis outside the CNS [59]. Thus, the tumor biology in GBM is entirely different compared to malignancies outside the CNS. The level of activity of circulating platelets and platelet-leukocyte conjugates has not been characterized in a well-defined group of GBM patients to date. Not only the disease, but its respective treatment (surgery, CTX, RTX) are risk factors for vascular events in cancer patients [5, 50, 60]. Of note, blood samples in the present study were obtained before any anticancer treatment. Besides systemic coagulative effects, platelets are in the focus to induce tumor-angiogenesis in several cancers [61–63]. A hallmark of GBM is a multitude of thrombosed vessels and platelets play a major role in this phenomenon [64–66] (Supplementary Figure 2). Distal of that thrombus hypoxia occurs, leading to angiogenesis. Platelets accumulate in a broad range of solid tumors and can be activated by direct cellular interactions with tumor cells via e.g. P-Selectin or the GPIIb/ IIIa [67–70]. Both receptors show an increased expression on platelets of GBM patients in the present study. A direct interaction between tumor cells and platelets in GBM could be a possible explanation for the finding of Nilson *et al,* who have shown EGVRvIII RNA in circulating platelets in GBM patients [71].

### Limitations of the study

Circulating platelets typically exhibit minimal levels of activation. Upon activation, the expression pattern of platelet surface receptors changes tremendously [10, 49, 72]. Measurement of platelet activation needs to be performed with caution to avoid pre-analytical artificial platelet activation [73]. In the present study, a median basal P-selectin expression proportion of 4.1% in the control group reflects an expected level under resting conditions as can be concluded from the literature [23, 24, 28]. This also indicated that an artificial platelet activation during sample acquisition was successfully avoided in our study. Matching of patients and the control study group was performed according to age and gender. Total platelet count did not differ between GBM and controls, as has been observed by other groups assessing platelet activation in cancer [27]. Brockmann *et al.* have shown that GBM patients with thrombocytosis have a worse prognosis, but only a minority of patients suffers from thrombocytosis [18]. In the present study none of the GBM patients presented with thrombocytosis, which is likely owed to the small study size. A correlation analysis (linear regression analysis) could not detect any correlation of the here investigated platelet activation parameters and the platelet count in the present study (Supplementary Figure 1).

Total leucocyte blood count was increased in GBM patients. However differentiation of leucocyte subtypes not available, making an interpretation of this finding difficult. The previous medical history was comparable between both groups. However, GBM patients received more often dexamethason (for the treatment of tumor edema), levitiracetam (for the treatment of symptomatic seizures), proton-pump-inhibition-therapy and thrombosis prophylaxis. All this symptomatic medication was given 1 to 5 days prior to blood sampling only, since study inclusion was at the time point of diagnosis. Furthermore, blood sampling was performed consistently in the early hours before administration of the morning medication to avoid circadian heterotopy and dexamethasone related effects [56, 74]. Thrombosis prevention with heparin is known to inhibit platelet P-selectin [75]. However, we have seen increased platelet P-selectin levels in GBM-patients. Thus we do not think that thrombosis prophylaxis did affect our results. Levitiracetam is known not to induce hematological changes in a short term treatment [76]. The matching process of GBM-patients and controls should address the above mentioned differences between both groups in future studies.

A further limitation of the study is that not all study parameters have been consistently analyzed in all GBM patients, since the study protocol had to be adjusted during patient recruitment. Nevertheless, most studies addressing platelet activation present data of *n* = 20 or even below. [20, 21, 23, 26, 28, 35, 36, 77–84]

## MATERIALS AND METHODS

### Patient selection and pre-analytical procedures

The study was approved by the local ethics committee and written informed consent was obtained from every study participant. Blood samples were obtained by a puncture of an antecubital vein at the day of admission of 27 consecutive patients with suspected glioblastoma multiforme (GBM) in the local department of neurosurgery. Blood sampling was down carefully and according to guidelines in order to avoid artificial platelet activation [73]. Hirudin (50 μg/ml) was used as anticoagulant in the platelet activation experiments to maintain a physiological calcium homoeostasis, which is crucial in the assessment of platelet activation markers [85]. Anticoagulation was done with sodium citrate (final concentration 109 mmol/l) in all other experiments. Patients underwent tumor resection or biopsy for histopathological diagnosis. 6 patients had to be excluded from the study, since the histopathological examination of the tumor tissue revealed another entity than GBM. Flow cytometric analysis was done immediately after blood sampling from the hirudin blood. Citrat anticoagulated blood was centrifuged into platelet rich plasma (PRP) and platelet poor plasma (PPP). PRP and PPP were immediately frozen at −80°C. The different n-counts for several study markers (P-selectin: *n* = 21, CD63: *n* = 19, CD40L: *n* = 12, Specific fibrinogen-binding: *n* = 11, platelet-leucocyte conjugates: *n* = 14, whole P-selectin: *n* = 19, thrombin generation assay: *n* = 19, S1P-level: *n* = 19) are due to a stepwise establishment of the study protocol. The study was registered at clinical trials.gov. In subsequent experiments, EDTA anticoagulated blood was sampled from 5 consecutive GBM patients as well as 5 age and gender matched controls. PBMC were gained by dense gradient centrifugation.

### Flow cytometry

Platelet surface markers for platelet detection (CD42b) and platelet activation (P-selectin, CD63, CD40L, specific fibrinogen binding to the activated GPIIb/IIIa) as well as the formation of heterotypic platelet-leucocyte conjugates and PSGL-1 on monocytes have been measured by flow cytometry [73].

### Sample preparation

Immediately after blood withdrawn PRP has been prepared and diluted 1:3 with Tyrodes solution to minimize artificial platelet aggregation and activation. For experiments with *in vitro* agonist induced platelet activation, either adenosine diphosphate (ADP, 50 μM, Sigma Aldrich, St. Louis, US) or the thrombin receptor-activating peptide (TRAP, 25 μM, Biosyntan, Berlin, Germany) were added. After incubation for 10 minutes at 37°C, samples were incubated with monoclonal antibodies or their respective isotype controls: anti-CD42b-APC (Biolegend, San Diego, US) as well as one of: anti-CD62-FITC (BD Pharmingen, Heidelberg, Germany), anti-CD63-FITC (BD Pharmingen, Heidelberg, Germany), anti-CD40L-FITC (BD Pharmingen, Heidelberg, Germany), Fibrinogen-FITC (Thermo Fisher Scientific, Waltham, US) or Fibrinogen-FITC+Tirofiban (Sigma Aldrich, St. Louis, US). Monoclonal antibodies have been concentrated equimolar to their respective isotypes as well as fibrinogen to tirofiban [86]. After incubation for 15 minutes in the dark, samples were diluted with PBS and flow cytometric analysis was done immediately.

For the quantification of PLC, monoclonal antibodies (mAb) anti-CD42b-APC (Biolegend, San Diego, US) and anti-CD45-PE (Biolegend, San Diego, US) or their respective isotype controls have been added to whole blood and incubated for 15 minutes in the dark. In samples with *in vitro* agonist platelet activation, a pre-incubation with either adenosine diphosphate (ADP, 50 μM) or the thrombin receptor-activating peptide (TRAP, 25 μM) for 10 minutes at 37°C was performed. After incubation of the mAb, erythrocytes were lysed with Lysing solution (BD Pharmingen, Heidelberg, Germany). After two washing steps, samples were diluted with PBS and flow cytometric analysis was done immediately [82].

For the quantification of PSGL-1 on the surface of monocytes, monoclonal antibodies CD14-APC-Vio770 (Milteny Biotec, Bergisch-Gladbach, Germany) and CD162-APC (Milteny Biotec, Bergisch-Gladbach, Germany) or their respective isotype controls have been added to PBMC and incubated for 15 minutes in the dark. Hereafter, samples were diluted with PBS and flow cytometric analysis was done immediately.

### Data analyses

Samples were measured by flow cytometry (Millipore Guava, Merck) and data analysis was done with the Guava software (Millipore Guava, Merck). Platelets were identified according to their forward and sideward light scatter characteristics and binding of the platelet specific anti-CD42b. Consecutive quantification of CD63, P-selectin, CD40L and Fibrinogen were done in the platelet population. Fluorescence labelled isotype-matched IgG antibodies and tirofiban were used to correct for non-specific binding of the mAb and fibrinogen, respectively. The percentage of positive platelets (CD63, CD62P, CD40L) or the median fluorescence intensity (MFI) (specific fibrinogen-binding) have been analysed.

Leucocytes (monocytes, granulocytes, lymphocytes) were identified according to their forward and sideward light scatter characteristics and binding of the leucocyte-specific anti-CD45. Consecutive quantification of CD42b was done in each leucocyte population. Fluorescence labelled isotype-matched IgG antibodies were used to correct for non-specific binding of the mAb. MFI of CD42b in the leucocyte populations was determined.

For detection of PSGL-1, monocytes were identified by their expression of CD14. Consecutive quantification of PSGL-1 was done in the monocyte population. Fluorescence labelled isotype-matched IgG antibodies were used to correct for non-specific binding of the mAb. MFI of PSGL-1 in the monocyte population was determined.

### Expression of whole circulating P-selectin

After thawing of frozen PRP and PPP samples, whole P-selectin determination was done by ELISA (Affymetrix, Santa Clara, US) according to the respective instructions. In brief, after washing the wells with buffer, sample diluent and respective samples or intrinsic controls were put into the wells. Every sample and control was measured in duplicate. HRP was added and incubation for two hours at room temperature on a microplate shaker took place. After washing the wells, TMB substrate solution was added. After incubation for 30 minutes in the dark, stop solution was added. The enzyme reaction was analyzed immediately after adding the stop solution with a spectro-photometer, 450 nm as the primary wave length and 620 nm as the reference wave length.

### Thrombin generation assay

The thrombin generation assay of thawed PRP and PPP samples was performed in a 96-well plate fluorometer (Fluoroskan Ascent). For each experiment two sets of readings were done, one from a well in which thrombin generation takes place (TG well) and a second one from a well to which the calibrator has been added (CL well). Each plasma sample has its own color which can influence the fluorescence intensity. Therefore each plasma sample needs to be compared to its own calibrator measurement. Typically, experiments were carried out in triplicates, i.e. a set of 2 TG wells is compared to a set of 2 CL wells. A dedicated software program (Thrombinoscope, Synapse BV, Maastricht, Netherlands) enabled the identification of the respective wells. To each well, 20 μl of plasma and 60 μl of Tyrode's solution were added. Afterwards 20 μl of the calibrator was added to the CL wells. In order to trigger the reaction, 20 μl of PPP reagent was added to the TG wells containing PPP. For the TG wells, which contain PRP, 20 μl of the PRP reagent was added to elicit the reaction. Afterwards, the plate was placed in the fluorometer and allowed to warm to 37°C (minimally 5 min). The dispenser added 20 μl of FluCa to all wells and this time point was registered as zero. During the measurement, the program compares the readings from the TG and the CL wells and directly calculates the thrombin concentration in a time dependent manner. Lag-time, overall thrombin generation and peak thrombin level were analyzed.

### Level of circulating plasma S1P

After thawing of frozen PRP and PPP samples, the level of S1P has been measured by liquid chromatography-tandem mass spectrometry as previously described [87]. In brief, 20 μL of PRP and PPP respectively were incubated with 20 μL of the internal standard (1 μM [16,17,18-^2^H_7_]-S1P (S1P-*d_7_*, Avanti Polar Lipids, Alabaster, AL, USA). Subsequently, proteins were precipitated with 350 μL of acetonitrile/water, 80/20, vol/vol. After centrifugation at 10,000 *g* for 15 min the extracts were subjected to reverse-phase chromatography on a Zorbax SB-C8 column (2.1 × 50 mm; Agilent Technologies, Santa Clara, CA, USA) at a flow rate of 0.35 mL/min. S1P was eluted with a binary gradient for 6 min (methanol/acetonitrile/0.1% formic acid, 2.5/2.5/95, vol/vol/vol to methanol/acetonitrile/0.1% formic acid, 30/30/40, vol/vol/vol) and measured by tandem mass spectrometry (Varian L1200 MS/MS, Agilent Technologies, Waldbronn, Germany) in the multiple reaction mode, monitoring the [M+H]^+^ S1P parent ion (m/*z* = 380) fragmentation to the daughter ion m/z = 264. The internal standard S1P-*d_7_* with the m/z 387 to 271 transition was used to correct for variations in sample preparation and instrument response. Calibration curves (four levels of S1P: 0; 0.1; 0.3; 1; 3 μmol/L) were generated to calculate absolute S1P concentrations in PRP and PPP samples.

### Statistics

Statistical analyses were performed with GraphPad Prism 5.0 (GraphPad Software, Inc., California, USA). Box plots are shown as the median and minimum to maximum. Pairwise comparisons were performed using Mann–Whitney *U* test. Statistical significance was defined as *p* < 0.05.

## CONCLUSIONS

The present study indicates for the first time an increased expression of platelet surface CD63 and soluble P-selectin serum levels in GBM patients compared to a matched control group. This is in agreement with the known elevated clinical risk for thrombosis in GBM. Despite enhanced platelet activation, the formation of heterotypic platelet-monocyte-conjugates was not increased. This may be due to the observed down-regulation of PSGL-1 on circulating monocytes in GBM patients. Whether this condition and the observed decreased plasma level of the immunomodulatory mediator S1P are indicative of a systemic anti-inflammatory status remains to be elucidated in future studies.

## SUPPLEMENTARY MATERIALS FIGURES



## Abbreviations

GBM: glioblastoma multiforme
CON: control
PLC: platelet-leucocyte-conjugates
PMC: platelet-monocyte-conjugates
DVT: deep vein thrombosis
RTX: radiation therapy
CTX: chemotherapy
S1P: sphingosine-1-phosphate
ETP: endogenous thrombin potential
TRAP: thrombin-receptor activation peptide
