# Supplementary material for: Platelet activation parameters and platelet-leucocyte-conjugate formation in glioblastoma multiforme patients
Source: Oncotarget. 2018 May 25;9(40):25860–76. doi: 10.18632/oncotarget.25395 (PMC5995223; doi:10.18632/oncotarget.25395)
Supplement: Supplementary file 1 [file oncotarget-09-25860-s001.pdf]

Platelet activation parameters and platelet-leucocyte-conjugate formation in glioblastoma multiforme patients

SUPPLEMENTARY MATERIALS

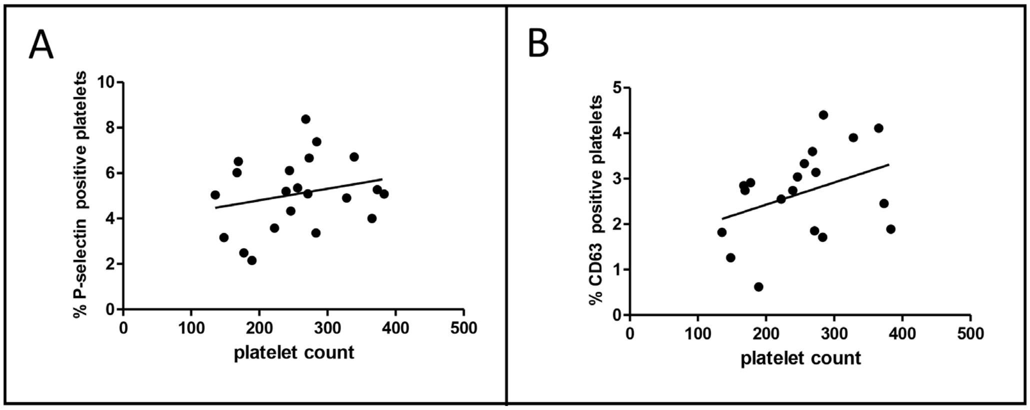

Figure 1: Correlation analysis (linear regression) of the total platelet count in the blood sample and the platelet surface expression of P-selectin (**A**) and CD63 (**B**) in GBM patients and control individuals.

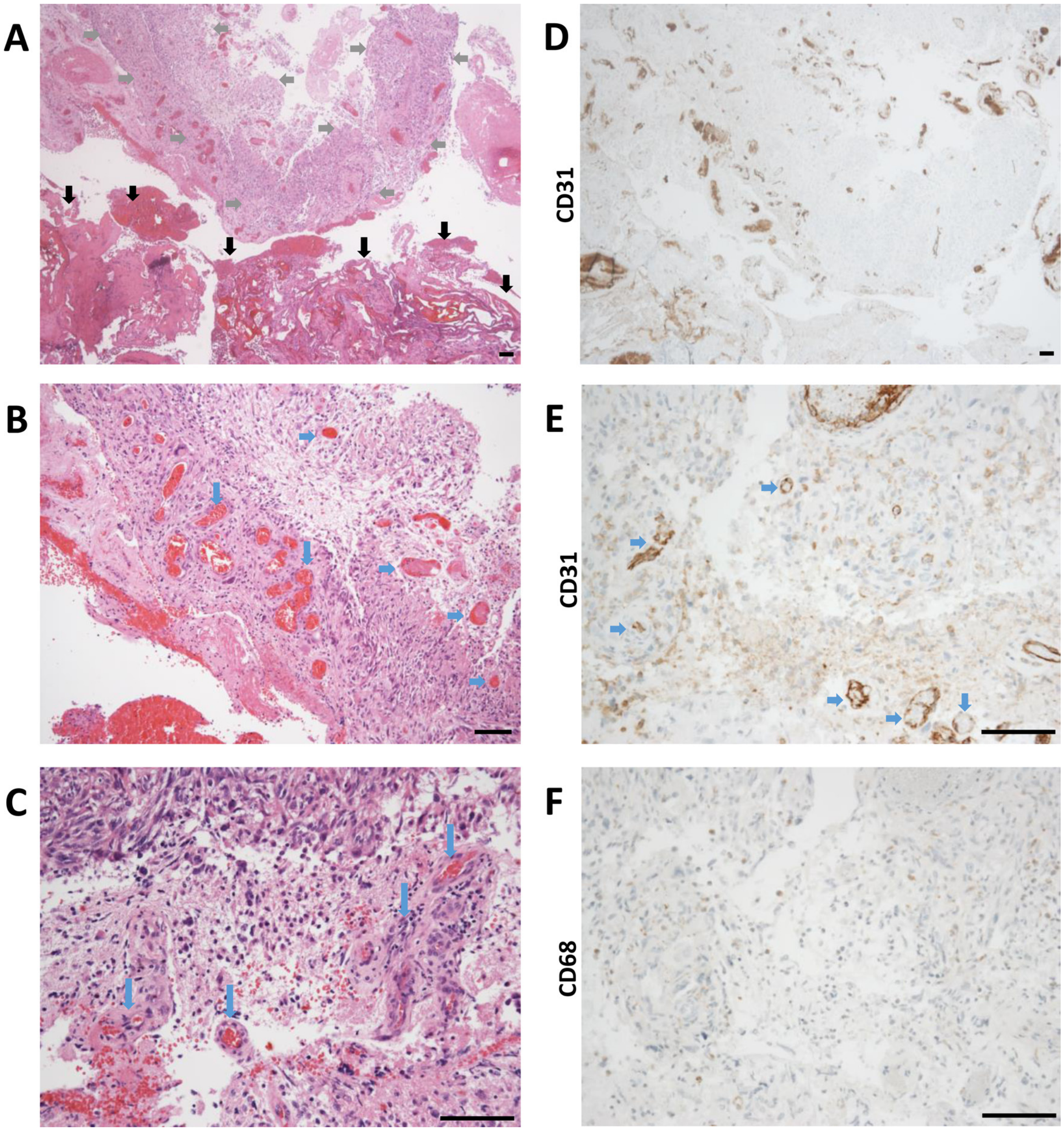

Figure 2: Micrographs illustrate typical fragmented glioblastoma specimen. (**A**) Grey arrows demarcate an area ofviable tumor tissue with prominent blood vessels; black arrows demarcate area of necrosis, hemorrhage, crush and thermal artefacts (H&E). (**B**) Blue arrows indicate intratumoral blood vessels (H&E) (**C**) Blue arrows indicate microvascular proliferations (H&E) (**D**) Immunohistochemical CD31 staining decorates blood vessels of different size sacross the specimen, including microvascular proliferations as highlighted in (**E**) by blue arrows. (**F**) Immunohistochemical CD68 staining decorates some macrophages (same area as shown in E). Scale bar = 100 μm.
